# MIR1246 in body fluids as a biomarker for pancreatic cancer

**DOI:** 10.1038/s41598-020-65695-6

**Published:** 2020-05-26

**Authors:** Fumitaka Ishige, Isamu Hoshino, Yosuke Iwatate, Satoshi Chiba, Hidehito Arimitsu, Hiroo Yanagibashi, Hiroki Nagase, Wataru Takayama

**Affiliations:** 1 Division of Hepatobiliarypancreatic Surgery, Chiba Cancer Center, Chuo-ku, Chiba Japan; 2 Division of Gastrointestinal Surgery, Chiba Cancer Center, Chuo-ku, Chiba Japan; 3 Laboratory of Cancer Genetics, Chiba Cancer Center Research Institute, Chuo-ku, Chiba Japan

**Keywords:** Gastrointestinal cancer, Cancer screening

## Abstract

Pancreatic cancer is an aggressive tumor associated with poor survival, and early detection is important to improve patient outcomes. In the present study, we examined MIR1246 expression as a biomarker of pancreatic cancer. Total RNA was extracted from serum, urine and saliva samples from healthy subjects (n = 30) and patients with pancreatic cancer (n = 41, stage 0–IV). The MIR1246 level in each fluid was analyzed by quantitative reverse transcription-polymerase chain reaction. Significantly higher MIR1246 expression in serum and urine was observed in patients with cancer than in healthy controls. A significant positive correlation was found between serum and urine MIR1246 expression (r = 0.34). Receiver operating characteristic curves were constructed for MIR1246 in all three body fluids. The area under the curve for serum MIR1246 was 0.87 (sensitivity, 92.3%; specificity, 73.3%), and that for urine MIR1246 was 0.90 (sensitivity, 90.2%; specificity, 83.3%). With a cut-off of the control group’s mean plus twice the standard deviation, the sensitivities of MIR1246 in serum and urine for pancreatic cancer were 60.9 and 58.5%, respectively. Combining both serum and urine MIR1246 expression yielded a sensitivity of 85%. These results indicate that MIR246 may be a useful diagnostic biomarker for pancreatic cancer.

## Introduction

Pancreatic cancer has an extremely poor prognosis. The 5-year survival rate based on data from 2008 to 2014 is only 8.5%. In the absence of lymph node or distant metastases from a lesion localized to the pancreas, the 5-year survival is 34.3%, indicating a better prognosis for early-stage disease. However, such cases of localized pancreatic cancer represent only 10% of all cases^1^ because symptoms often do not appear until the disease has progressed^2^. The development of screening tests facilitating early detection and treatment is presently considered the most important strategy for improving the outcomes of pancreatic cancer.

The main tumor markers for pancreatic cancer are serum levels of carbohydrate antigen 19-9 (CA19-9), carcinoembryonic antigen (CEA) and duke pancreatic monoclonal antigen type 2 (DUPAN2), which have sensitivities for pancreatic cancer of 70–80%, 30–60% and 50–60%, respectively^3,4^. However, these markers are less likely to be positive until pancreatic cancer reaches an advanced stage. In addition, the false-positive rate of CA19-9 is relatively high at 20–30%^5^. One report noted that CA19-9 is positive in only 48.4% of cases when the pancreatic tumor is ≤2 cm in size^6^,, and thus, it is not useful for the early diagnosis of this cancer.

MicroRNAs are short non-coding RNAs composed of 18–25 nucleotides. In 2001, microRNAs were recognized as evolutionarily conserved sequences in many organisms, including humans^7^. Aberrant microRNA expression has been demonstrated in various malignancies, and it is considered to be associated with carcinogenesis and progression^8–11^. MicroRNAs circulate in a cell-free form in blood^12–14^, and many studies have demonstrated the diagnostic and prognostic utility of circulating microRNAs in patients with cancer.

Previously, we reported the potential usefulness of MIR1246 as a biomarker for esophageal cancer^15^. It has been suggested that MIR1246 functions as part of the p53-related cell-to-cell network, and it is related to chemoresistance and cancer stem cell-like properties in pancreatic cancer^16,17^. Additionally, several reports identified elevated levels of MIR1246 expression in urine and saliva in patients with pancreatic cancer^18–20^.

However, to the best of our knowledge, no reports verified the elevated expression of microRNAs in different types of body fluid in the same subjects. In the present study, we evaluated the expression of MIR1246 in serum, urine and saliva in patients with pancreatic cancer and examined its potential as a biomarker for pancreatic cancer.

## Material and Methods

### Specimens

Between April 2017 and November 2019, venous blood, urine and saliva samples were collected from 41 patients with pancreatic cancer and 30 healthy controls at the Chiba Cancer Center (Chiba, Japan), although saliva samples could only be obtained from 22 patients with pancreatic cancer. The samples were collected prior to any treatment, including surgery, chemotherapy, or radiation. In addition, in the group of patients with pancreatic cancer, serum CA19-9 and CEA levels were tested before treatment.

The venous blood samples were centrifuged at 1580 ×*g* for 5 min at 4 °C to obtain serum. Urine collection was performed when convenient for the patient. Urine was centrifuged (1580 ×*g*, 5 min, 4 °C) to obtain a supernatant. Saliva collection was performed after an oral rinse at some time other than after a meal. Saliva was centrifuged (1580 ×*g*, 5 min, 4 °C) to obtain a supernatant. If separation was incomplete, centrifugation was repeated for another 5 min. The samples were then stored at −80 °C until further processing. Written informed consent for inclusion in the study was obtained from each patient. The study was approved by the Ethics Committee of Clinical Research at Chiba Cancer Center (No. H29-0005) and conducted in compliance with the Declaration of Helsinki.

### RNA extraction

Total RNA was extracted from 200 μl of serum, urine and saliva samples using an miRNeasy Serum/Plasma Kit (QIAGEN, Hilden, Germany) according to the manufacturer’s instructions. In addition, MIR39 contained in the kit was used as a spike-in control.

### cDNA generation by reverse transcription

Total RNA was reverse-transcribed to cDNA using a miScript II RT Kit (QIAGEN). From the obtained RNA solution, 12 μl of template RNA having a concentration of 4.17 ng/μl were prepared. The reverse transcription master mix was created by mixing 4 μl of 5× miScript HiSpec Buffer, 2 μl of 10× miScript Nucleics Mix and 2 μl of miScript Reverse Transcriptase Mix. The template RNA was added to the tube containing the reverse transcription master mix. The solution was incubated for 60 min at 37 °C. It was then further incubated for 5 min at 95 °C to inactivate miScript Reverse Transcriptase Mix and placed on ice.

### Quantitative reverse transcription-polymerase chain reaction (qRT-PCR)

qRT-PCR was performed using the miScript SYBR Green PCR Kit (QIAGEN). The following the sequence-specific forward primers were used: MIR1246, 5′-AAUGGAUUUUUGGAGCAGG-3′; and MIR39, 5′-UCACCGGGUGUAAAUCAGCUUG-3′.

PCR was performed in a 7300 Real-Time PCR system (APPLIED BIOSYSTEMS, Foster City, CA, USA) using an amplification program of 95 °C for 15 min followed by 40 cycles of 94 °C for 15 s, 55 °C for 30 s and 70 °C for 34 s). All reactions were performed in duplicate. The 2^−ΔCt^ method was used to calculate the relative expression of MIR1246 (ΔCt = Ct [MIR1246] − Ct [MIR39]).

### Statistical analysis

Conformation of the data to a normal distribution was determined using Shapiro–Wilk’s test. Based on the results, the statistical methods were selected as follows. An unpaired Student’s *t*-test was performed to compare differences in age. Wilcoxon’s signed-rank test was performed to compare differences in MIR1246 expression between patients with cancer and healthy controls. Spearman’s rank correlation coefficient was used to assess the correlation of MIR1246 expression among the three body fluid samples. The χ2 test or Fisher’s exact probability test was used to evaluate the correlation between serum or urine miRNA expression and clinicopathologic tumor factors. Receiver operating characteristic (ROC) curves and area under the curve (AUC) were used to assess the sensitivity and specificity of serum/urine/saliva miRNA expression and CA19-9/CEA for detecting pancreatic cancer. The optimal cut-off values in ROC curves was set to the value that maximizes the Youden index. The Youden index was defined as sensitivity + specificity − 1. All tests were two-sided, and the significance level was set at p < 0.05. JMP 14 (SAS INSTITUTE INC., Cary, NC, USA) software was used for the analyses.

## Results

### Subjects

All stages of pancreatic cancer (Stage 0–IV, the seventh edition of UICC TNM classification) were observed in the study group, and 24 of 41 patients had undergone tumor resection (Table 1). Fourteen patients had distant metastasis, and three patients had an unresectable primary lesion. The tumor types included pancreatic ductal carcinoma (n = 36), IPMN with high-grade dysplasia (n = 2), IPMN-associated pancreatic cancer (n = 2) and anaplastic carcinoma (n = 1). The median observation period for these cases was 306 days (30–679).

**Table 1:** The clinical characteristics of 41 PC patients and 30 healthy controls.

	PC patients	Healthy controls	p
Number	41	30	
Male/Female	26/15	21/9	1.00
Age (range)	68.1 (48–89)	62.5 (51–76)	0.028
UICC TNM			
0/IA/IB	2/1/1		
IIA/IIB	5/16		
III/IV	2/14		
Primary tumor			
Tis/T1/T2	2/1/1		
T3/T4	29/8		
Lymph node metastasis			
N0/N1	10/31		
Distant metastasis			
M0/M1	27/14		
Surgical resection	24		
Pancreaticoduodenectomy	19		
Distal pancreatectomy	3		
Total pancreatectomy	2		
Cancer type			
PDAC	36		
IPMN with high-grade dysplasia	2		
IPMN-associated PC	2		
anaplastic carcinoma	1		

Abbreviation: IPMN, intraductal papillary mucinous neoplasm PC, pancreatic cancer; PDAC, pancreatic ductal adenocarcinoma.

### MIR1246 expression

**Figure 1:**
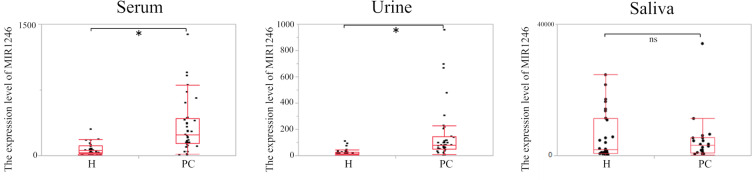
Expression of MIR1246 in serum, urine and saliva in healthy controls and patients with pancreatic cancer. H: healthy controls, PC: pancreatic cancer, *p < 0.0001, ns: p > 0.05.

**Figure 2:**
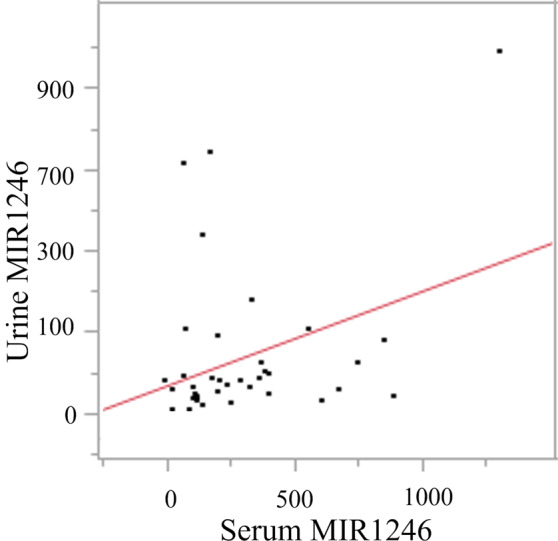
Correlation of MIR1246 expression between serum and urine.

MIR1246 expression in serum and urine was significantly higher in patients with pancreatic cancer than in the healthy controls (p < 0.0001), but the levels in saliva did not differ significantly between the two groups (p = 0.76; Fig. 1). A significant positive correlation was found between serum and urine MIR1246 expression (r = 0.34; p = 0.033, Fig. 2) but not between serum and saliva MIR1246 expression or between urine and saliva MIR1246 expression.

### Diagnostic ability of MIR1246 to differentiate patients with pancreatic cancer from healthy controls

**Figure 3:**
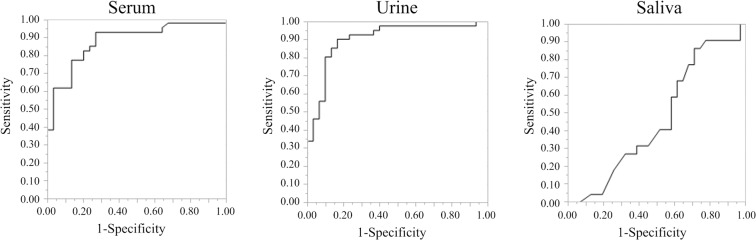
Receiver operating characteristic curves of MIR1246 levels in serum, urine and saliva.

ROC curves were constructed for MIR1246 expression in serum, urine and saliva to compare their diagnostic value for pancreatic cancer (Fig. 3). The AUC for serum MIR1246 expression was 0.87 (cut-off value, 71.1; sensitivity, 92.3%; specificity, 73.3%), and that for urine MIR1246 was 0.90 (cut-off value, 25.4; sensitivity, 90.2%, specificity, 83.3%), indicating good diagnostic accuracy. The AUC for saliva MIR1246 expression was 0.48 (cut-off value, 11271.7; sensitivity, 91%; specificity, 26.7%). Similarly, ROC curves were constructed for serum CA19-9 and CEA (see Supplementary Fig. S1). The AUC for serum CA19-9 was 0.89 (cut-off value, 14.5; sensitivity, 85.0%; specificity, 93.1%), and that for serum CEA was 0.80 (cut off value, 4.5; sensitivity, 60.0%; specificity, 93.1%).

**Figure 4:**
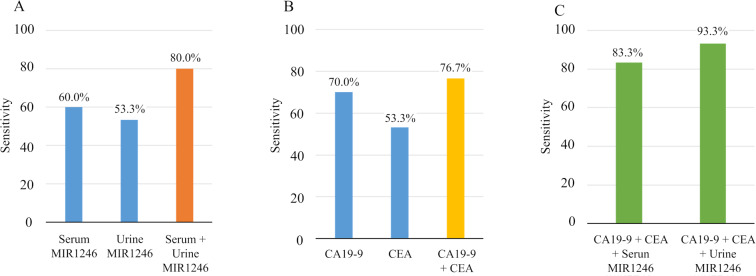
(**a**) The sensitivity of MIR1246 expression in serum and urine and the combination of both serum and urine MIR1246 expression. (**b**) The sensitivity of serum carbohydrate antigen 19-9 (CA19-9), carcinoembryonic antigen (CEA), and their combination. (**c**) The sensitivity of the combination of serum CEA and CA 19-9 and MIR1246 expression in serum or urine.

Based on the mean MIR1246 expression in the control group, the cut-off value was defined as the control mean plus twice the standard deviation. Using this cut-off value, the sensitivity of serum and urine MIR1246 expression were 60.9% and 51.2%, respectively. The sensitivity for combined serum and/or urine MIR1246 expression was 80.5% (Fig. 4a). The corresponding sensitivity for serum CA19-9 and CEA expression were 68.3% and 51.2%, respectively, and the sensitivity of CA19-9 or CEA expression was 80.5% (Fig. 4b). The sensitivity for combined CA19-9, CEA and/or serum MIR1246 expression was 92.6%, and combining CA19-9 and CEA with urine MIR1246 yielded a sensitivity of 87.8% (Fig. 4c). Thus, adding either serum or urine MIR1246 to the combination of CA19-9 and CEA improved the sensitivity, although the difference was not statistically significant (p = 0.317 and p = 0.071).

### Correlation between serum or urine MIR1246 expression and clinicopathologic features of pancreatic cancer

We examined possible associations between serum and urine MIR1246 expression and the clinicopathologic features of pancreatic tumors. High and low levels were defined using the optimal cut-off value obtained from the ROC curve.

Serum and urine MIR1246 levels displayed no correlations with tumor invasion, lymph node metastasis, distant metastasis, or cancer stage (Table 2). It should be noted that analysis of tumor invasion and lymph node metastasis was performed only for the 24 patients for whom the lesion had been resected and a detailed pathology report was available.

**Table 2:** The correlation between the serum or urinary MIR1246 expression and clinicopathological factors of PC patients.

Characteristics	*n*	High serum MIR1246 expression	Low serum MIR1246 expression	*P* value	High urinary MIR1246 expression	Low urinary MIR1246 expression	*P* value
Tumor depth							
T0-2	4	4	0	**1.0**	2	0	**1.0**
T3–4	20	19	1		18	2	
Lymph node metastasis							
N0	9	9	0	**1.0**	8	1	**1.0**
N1	15	14	1		14	1	
Distant metastasis							
M0	27	26	1	**0.10**	14	13	**0.31**
M1	14	11	3		4	10	
Stage							
0-IIA	9	9	0	**0.56**	8	1	**1.0**
IIB-IV	32	28	4		28	4	

## Discussion

In this study, we compared the expression of MIR1246 in blood, urine and saliva between patients with pancreatic cancer and healthy controls. Significant differences in MIR1246 levels in serum and urine were noted between patients with cancer and the healthy controls, but the saliva MIR1246 levels did not differ significantly between the groups. These results indicate that MIR1246 expression in serum and urine may prove useful as a pancreatic cancer biomarker.

MIR1246 is considered an oncomiR in various cancer types^21^. The potential use of MIR1246 as a biomarker for diagnosis has been reported for hepatocellular carcinoma (HCC)^22^, ovarian cancer^23^ and esophageal cancer^15^. Several groups investigated the function of the intercellular MIR1246 network. In HCC cell lines, MIR1246 was found to decrease cell adhesion molecule 1 (CADM1) expression, thereby enhancing cell migration and invasion. CADM1 is a well-defined tumor suppressor gene that was discovered recently^24^. Another study found that octamer 4 activates MIR1246 expression, leading to an increase in the stemness of liver cancer cells^25^. In addition, in pancreatic cancer cells as well as a mouse model, MIR1246 expression was associated with cancer cell stemness and chemoresistance by targeting cyclin G2 (CCNG2). CCNG2 is also known as a tumor suppressor gene, and it has been reported to be downregulated in various cancers^17^.

Several studies examined microRNA levels in the plasma and serum of patients with pancreatic cancer and reported the efficacy of MIR1246 for diagnosis^18,26^. However, obtaining blood requires a needle prick, whereas urine and saliva can be obtained noninvasively. Hence, they may be more useful than serum for screening tests. Urine MIR30e, MIR143, MIR223 and MIR204 levels have proven useful as biomarkers of pancreatic cancer^19^.

Because urine is a filtrate of blood, it was hypothesized that the types and amounts of microRNAs in the two fluids might be similar. We found that MIR1246 expression in serum and urine was positively correlated. MicroRNAs are encapsulated in exosomes (diameter, 40–100 nm) and extracellular vesicles (such as microvesicles and apoptotic bodies) with large diameters, and they are secreted from cells to the extracellular space^27^. Furthermore, there are reports demonstrating that extracellular vesicles circulating in the blood can be transferred into urine in mice^28,29^. Meanwhile, a study of microRNAs from various fluids in healthy individuals were comprehensively analyzed using next-generation sequencing, and the expression patterns were found to differ between serum and urine^30^. Urine contains extracellular vesicles derived from the tubular epithelium and glomerular cells^31–33^. Therefore, the types and amounts of microRNAs expressed in urine and blood may differ, even in the same subject.

Some studies examined the levels of microRNA in the saliva of patients with pancreatic cancer^34,35^. Saliva is also a blood-derived fluid that is believed to contain most of the molecules present in blood^36^. Therefore, it may be possible to monitor changes in such substances in the blood indirectly using saliva instead of serum or plasma^34,37^.

Xie *et al*. reported that the combination of MIR3679-5p and MIR940 in saliva was effective as a biomarker for pancreatic cancer. The sensitivity and specificity of the combination were 72% and 70%, respectively^34^. Alemar *et al*. reported significantly higher serum MIR21 and MIR34a levels in serum from patients with pancreatic cancer than in healthy controls, but the levels in saliva did not differ significantly^35^. Thus, as found in the present study, serum and saliva microRNA levels may differ from each other.

The ROC curves based on the data obtained in the current study revealed the relatively high diagnostic ability of serum and urine MIR1246 for pancreatic cancer. And the ability was comparable to that of existing tumor markers, and the sensitivity was slightly higher. Nonetheless, they did not reach the level required for screening tests for this disease. For example, the fecal occult blood test is a well-known screening test for colorectal cancer, with a reported sensitivity and specificity of as high as 83 and 96%, respectively^38^. Studies also indicated that program sensitivity is further increased by performing several fecal occult blood tests during the detectable pre-clinical phase^39^. By contrast, the incidence of pancreatic cancer is approximately one-fourth that of colorectal cancer, and it is a relatively fast-growing neoplasm. For this reason, screening tests for pancreatic cancer may require higher sensitivity and specificity.

One of the limitations of this study is the relatively small number of cases, especially in the early stages. The sensitivity of CA19-9 and CEA for localized pancreatic cancer cases (stage 0-IIA, n = 9) were both 33.3%, while the sensitivity of serum and urine MIR1246 were 100% and 88.9%, respectively (data not shown). However the number of cases of early-stage disease in this study was too small to conclude that MIR1246 is useful for early diagnosis. A greater number of such cases must be accumulated to confirm our findings. Another limitation of this study is that it focuses on a single microRNA. It is unlikely that a single microRNA can be diagnostic for all cancers. Various genes, such as KRAS, TP53, cyclin-dependent kinase inhibitor 2 A, SMAD4 and breast cancer susceptibility genes 1 and 2, are known to be associated with the development and progression of pancreatic cancer^40^. In addition, any particular microRNA may downregulate a large number of mRNAs^41^. Therefore, several types of microRNAs highly expressed in precancerous and cancerous states are likely to circulate in body fluids. A comprehensive analysis of body fluids such as serum and urine using next-generation sequencing is needed to identify multiple microRNAs as biomarker candidates that must then be validated in large numbers of cases.

## Supplementary information

Supplementary Figure S1
