# Supplementary figures and images for: MIR1246 in body fluids as a biomarker for pancreatic cancer
Source: Sci Rep. 2020 May 26;10:8723. doi: 10.1038/s41598-020-65695-6 (PMC7250935; doi:10.1038/s41598-020-65695-6)

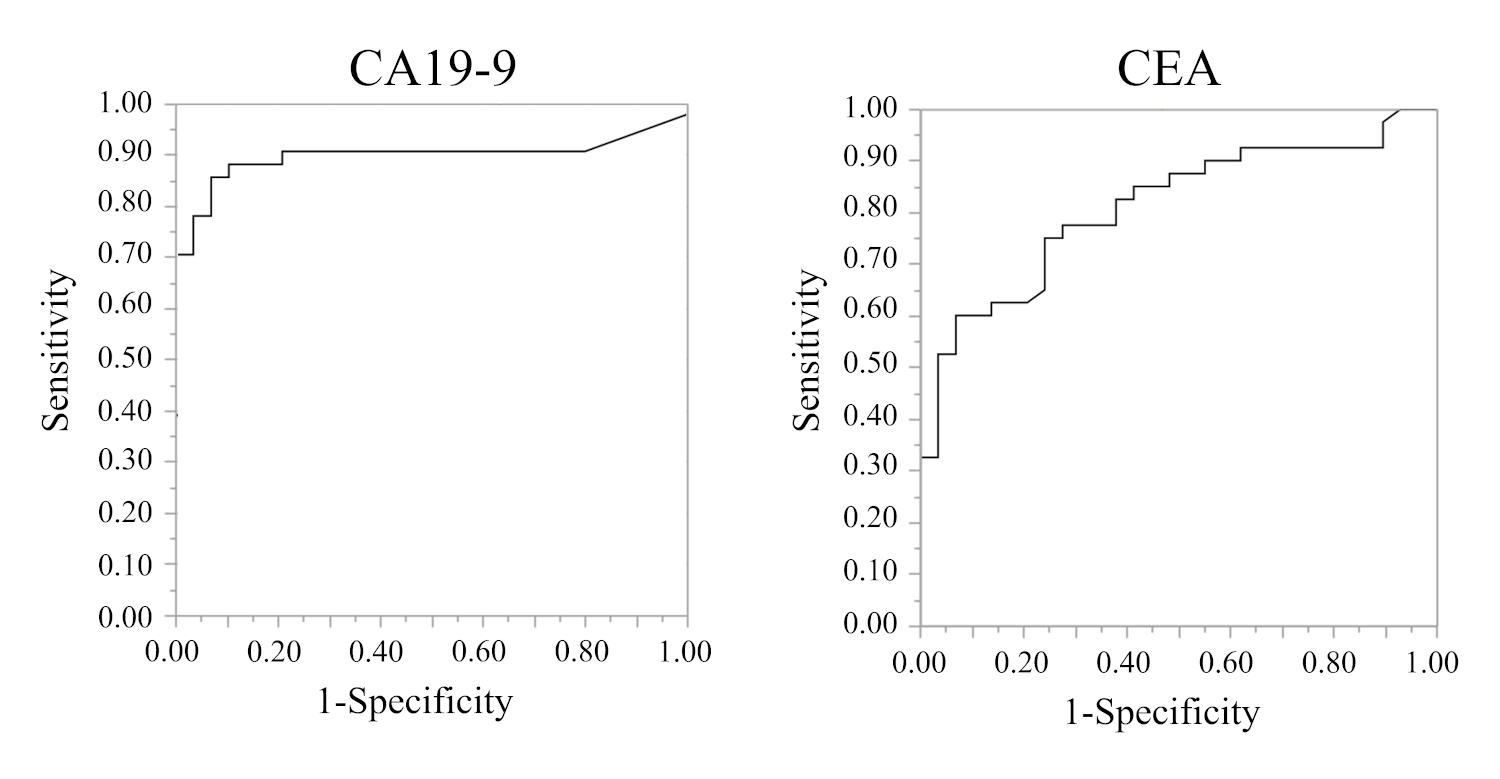

Supplement: Supplementary file 1 — Supplementary Figure S1 [file 41598_2020_65695_MOESM1_ESM.tiff]
